# A Preliminary Report for the Design of MoS (Micro-Olive-Spreadsheet), a User-Friendly Spreadsheet for the Evaluation of the Microbiological Quality of Spanish-Style Bella di Cerignola Olives from Apulia (Southern Italy)

**DOI:** 10.3390/foods9070848

**Published:** 2020-06-29

**Authors:** Antonio Bevilacqua, Barbara Speranza, Daniela Campaniello, Milena Sinigaglia, Maria Rosaria Corbo

**Affiliations:** Department of the Science of Agriculture, Food and Environment, University of Foggia, 71122 Foggia, Italy; antonio.bevilacqua@unifg.it (A.B.); barbara.speranza@unifg.it (B.S.); daniela.campaniello@unifg.it (D.C.); milena.sinigaglia@unifg.it (M.S.)

**Keywords:** table olives, Bella di Cerignola, brines, microbiological quality, user-friendly spreadsheet, producers

## Abstract

A user friendly spreadsheet (Excel interface), designated MoS (Micro-Olive-Spreadsheet), is proposed in this paper as a tool to point out spoiling phenomena in Bella di Cerignola olive brines. The spreadsheet was designed as a protected Excel worksheet, where users input values for the microbiological criteria and pH of brines, and the output is a visual code, much like a traffic light: three red cells indicate a spoiling event, while two red cells indicate the possibility of a spoiling event. The input values are: (a) Total Aerobic Count (TAC); (b) Lactic Acid Bacteria (LAB); (c) yeasts; (d) staphylococci; (e) pH. TAC, LAB, yeasts, and pH are the input values for the first section (quality), while staphylococci count is the input for the second section (technological history). The worksheet can be modified by adding other indices or by setting different breakpoints; however, it is a simple tool for an effective application of hazard analysis and predictive microbiology in table olive production.

## 1. Introduction

The olive tree is an iconic species in Mediterranean cultural history and diet. Its multiple uses in the food industry (olive oil and table olives) and its omnipresence in many traditional agro-systems have made this species an economic pillar and cornerstone of Mediterranean agriculture [1]. Its role as a symbol of Italy, in particular of the Region of Apulia, has increased due to the emergence of the *Xylella* outbreak. Table olives represent one of the most popular fermented foods in the Mediterranean basin and in Italy, but their production is increasing worldwide, as suggested by the International Olive Council (IOC) statistics for the period 2013-14/2017-18, with a duplication of amounts produced compared to the beginning of the millennium [2]. European Union (EU) covers 31% of world production, with Spain, Italy, and Greece being the major producing countries (97% of EU production) [2]. The olive fruit cannot be consumed directly because of the presence of oleuropein. The bitterness can be removed by alkaline treatment, or by brining/salting, fermentation, and acidification [3]. The trade standard applying to table olives describes the type of preparation of table olives that are treated, natural olives, dehydrated and/or shriveled olives, and olives darkened by oxidation; however, some traditional processes are still applied, such as the Castelvetrano system [3].

In Italy, table olive production is mainly located in Southern regions (Apulia, Sicily, etc.); however, fermentation still relies upon natural microbiota [4]. The three main techniques for table olive production used in Italy concern 82% green olives, 16% black olives and 2% processed at the cherry ripened stage [5].

The microbial ecosystem is complex and can be affected by several intrinsic (pH, water activity, diffusion of nutrients from the drupe, and concentration of phenols) or extrinsic factors (temperature, oxygen availability, and salt) [6]; if these variables are not controlled, a microbial spoilage could occur. In traditional fermentations, the modulation of salt and pH is the only way to counteract spoilage. Lactic acid bacteria (LAB) and yeasts represent the microbiota normally involved in fermentation [4], but members of *Staphylococcus* and *Pseudomonas* are detected at the beginning and throughout the process [7,8,9], along with some pathogens, such as *Clostridium botulinum*, *Listeria monocytogenes*, and *Staphylococcus aureus* [10,11,12,13]. Enterobacteriaceae can be also found at the beginning of fermentation and are quickly inhibited by the pH decrease by LAB [14]. LAB species encountered during table olive fermentation are *Lactiplantibacillus plantarum*, *L. pentosus*, and to a lesser extent, *L. paraplantarum* [5]. They are responsible for the rapid and safe acidification of brines [5]. Besides, different yeast species are recovered, such as *Wickerhamomyces anomalus*, *Pichia membranifaciens*, *Saccharomyces cerevisiae*, *Debaryomyces hansenii,* and *Candida boidinii* [5]. 

In Italy the most important varieties for table olive production are, among others, Bella di Cerignola [15], Nocellara Etnea [4], Tonda di Cagliari [16], Giarraffa [17], Termite di Bitetto, Cellina di Nardò [18], and Leccino [19], treated by either Spanish or natural styles. Bella di Cerignola (formerly Bella della Daunia, variety Bella di Cerignola, Protected Denomination of Origin-PDO) is one of the most important variety of olives in the region of Apulia. It is processed through Spanish and natural styles between October and December [8]. The fermentation of table olives still relies on natural processes led by the indigenous microbiota from the raw materials (olives, salt, water) or that are acquired during processing at factory facilities (fermenters, tanks, pipelines, pumps) and fermenter yards [20]. However, a part of the microbial diversity associated with this fermentation has been “domesticated” by the continuous replication of peculiar processing conditions and know-how. This might represent a significant contribution to “terroir” aspects [20].

The production of table olives from Bella di Cerignola through Spanish style relies upon a three-step protocol, similar to that reported by Mastralexi et al. [2] for Greek PDO table olives “Prasines Elies Chalkidikis”, with some differences in the duration of each step, such as the amount of salt and lye. The three steps are as follows: (a) sorting and size grading, debittering and neutralization; (b) brining (6–10% NaCl) and fermentation (ca. 2 months); (c) storage in plastic tanks filled in brine until packaging and thermal treatment. Traditionally, fermentation is divided into four steps or phases [21]; in the first phase, Gram negative bacteria prevail, and pH decreases from 9.0 to around 6.0. This phase lasts until the growth of LAB (normally 48–72 h). The second and the third steps are characterized by the growth of LAB, along with the strong acidification of the brine. At the end of this third step, there is a potential fourth step, characterized by an increase in pH and volatile acidity, and a decrease in lactic acid [21]. The duration of this last step, usually referred to as the post-fermentation stage, is quite variable (from a month to a year), and depends on demand and market prices. If pH and NaCl are not strictly controlled, a microbial spoilage can occur due to a variety of microorganisms (*Aerobacter*, bacilli, propionibacteria, oxidative yeasts, moulds, etc.) [3,5,21]. Although new trends have been exploited in table olive production (the use of probiotics, the combinations of yeasts and LAB as starter cultures, low-salt fermentations, the study of a biogeography of olives, models to optimize the amounts of preservatives, the use of starter cultures able to degrade oleuropein) [3,22], to the best of our knowledge there are no user-friendly tools used in the post-fermentation stage in order to control the microbiological quality of batches and to help producers to perform corrective strategies.

Therefore, this paper represents a first approach to design a user-friendly worksheet that can be used by producers of Bella di Cerignola olives as a tool to focus on the microbiological stability of olives during their storage in tanks before pasteurization. The specific aims of this research were: (a) to link the idea of spoiled samples from olive producers to olive microbiology; (b) to design a simple quality management tool requiring few data; (c) to validate the tool.

## 2. Materials and Methods 

### 2.1. Samples

Olives (cv. Bella di Cerignola) and brines from Spanish style processing were collected after the fermentation and/or olive storage in tanks during winter and spring from four different factories located in Cerignola (Foggia County, Southern Italy). Eighty-nine different samples were analyzed to assess microbiota, pH, NaCl amounts (brines) and sensory scores (olives); of which, 77 samples were used to design the user-friendly worksheet, and 12 for the validation.

### 2.2. Microbiological Analyses

Olive brines were serially diluted in saline solution (0.9% NaCl) and plated on the following media: (a) Plate Count Agar (PCA), incubated at 30 °C for 24–48 h for total aerobic count (TAC); (b) PCA, incubated at 30 °C for 24–48 h, after a heat-shock of dilutions at 80 °C for 10 min (aerobic spore-forming bacteria); (c) MRS agar, supplemented with 0.17 g/L cycloheximide (Sigma-Aldrich, Milan, Italy), incubated at 30 °C under anaerobic conditions for 48–72 h for lactic acid bacteria (LAB); (d) Pseudomonas Agar Base, added to CFC selective supplement (containing the cetrimide), incubated at 25 °C for 48–72 h for pseudomonads; (e) Baird Parker Agar Base, added with Egg Yolk Tellurite Emulsion (37 °C for 24–48 h) and Mannitol Salt Agar (37 °C for 24–48 h) for staphylococci; (f) Violet Red Bile Glucose Agar (VRBGA), incubated at 37 °C for 18–24 h for enterobacteria; (g) SPS Agar, incubated under strict anaerobic conditions at 37 °C for 24 h for clostridia; (h) Sabouraud Dextrose Agar, supplemented with 0.1 g/L chloramphenicol (C. Erba, Milan), incubated at 25 °C for 2–4 days for yeasts. All media and supplements were purchased from Oxoid (Basingstoke, UK) [8,23].

### 2.3. pH and NaCl Amount

The pH of brines were measured using a pH-meter Crison (Crison Instruments, Barcelona, Spain), whereas salt amounts were evaluated by means of refractometer Sper Scientific model 106 ATC (Scottsdale, AZ, USA).

### 2.4. Sensory Score

The producers were asked to analyze olives and brines; each producer analyzed his own samples. Before sensory evaluation, a meeting with all producers was done in order to define the sensory properties of Bella di Cerignola olives and what they meant by “good quality”. The focus of this meeting was to document the “Sensory Analysis of Table Olives” [24] and the definition of negative defects (abnormal fermentation, musty, rancid, cooking effect, soapy, metallic, earthy, winery/vinegar), along with the attributes of olives (acid, bitterness, salty taste, hardness, fibrousness, crunchiness of texture).

The output of this consensus panel was to define “spoiled” samples as those with at least one of the negative attributes or if the other attributes showed negative changes. Producers were asked to score (0 vs. 1) odor, color, taste, and finally to give a score of 1 (spoiled) or 0 (non-spoiled); in case of doubts the output could be “acceptable with some problems (spoilage is beginning)”. Scores were confirmed by five researchers of the laboratory of Predictive Microbiology, usually consuming green table olives.

### 2.5. Data Analysis

Microbiological count, pH, and salt analyses were repeated twice for each batch, and all results were analyzed through *t*-test (*p* < 0.05) using the software, Statistica for Windows (Statsoft, Tulsa, OK, USA).

### 2.6. Spreadsheet Design

The spreadsheet (MoS, Micro-Olive-Spreadsheet) was designed as a protected Excel spreadsheet, where users can input values for microbiological criteria and the pH of brines (Spreadsheet S1, Supplementary Materials). The input values are: (a) Total Aerobic Count (TAC); (b) Lactic Acid Bacteria (LAB); (c) yeasts; (d) staphylococci; (e) pH. TAC, LAB, yeasts, and pH are the input values for the first section (quality), while staphylococci count is the input for second section (technological history). Microbial concentrations refer to the values of the brines, as log CFU/mL (decimal logarithm).

The spreadsheet is designed based on four different cells with “If” functions. The possible outputs of “If” are “1” (true or yes) or “0” (false or no). The functions are:

Quality

- LAB < 1% TAC. The function was evaluated through the exponential values of cell count, rather than with a logarithm;
- Yeast > 5;
- pH > 4.5.

Technological process

- Staphylococci > 4.

Salt

- salt<8%.

In the cells containing the output of the “If” function, there is a conditional formatting linked to the output: if the results of function are true (or 1), the cells becomes red, while if the results are false (0) the cells become green.

## 3. Results

### 3.1. Data

**Figure 1:**
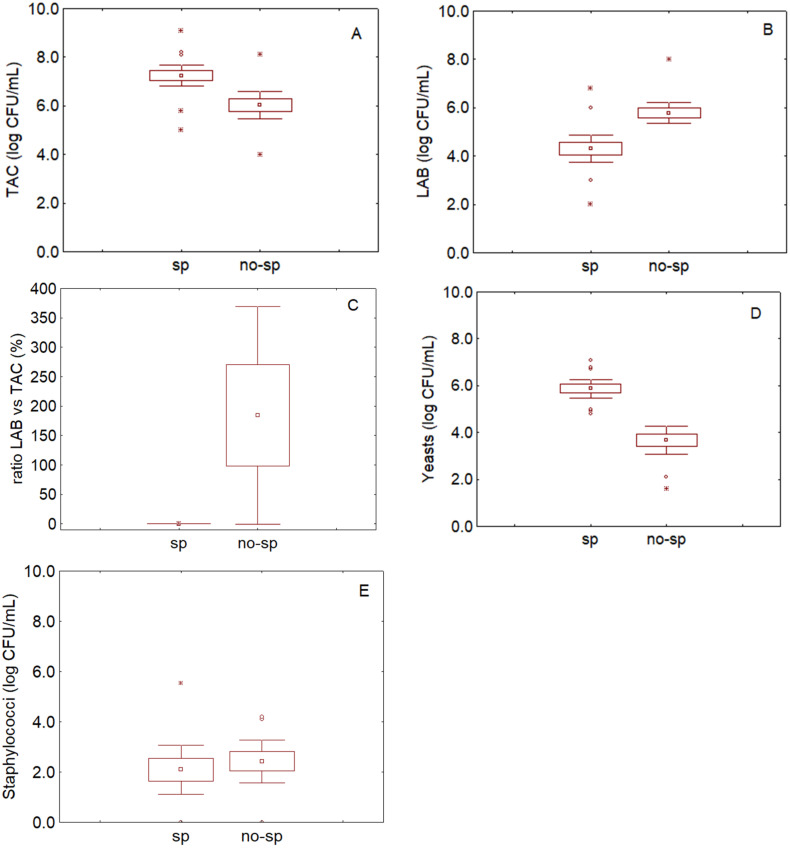
Total Aerobic Count (TAC) (**A**), Lactic Acid Bacteria (LAB) (**B**), ratio LAB vs. TAC (**C**), yeasts (**D**), and staphylococci in brines (**E**). sp, spoiled samples, no-sp, non-spoiled samples. ▫ Mean; □, Mean ± SE; ◦ Outliers; * extremes; bars denote 95% confidence interval.

Seventy-seven samples were analyzed in the first step; 12 were randomly selected and used for the validation. First, the samples were analyzed by producers and researchers to cluster them in two groups: spoiled and non-spoiled. In case of doubts (different clustering between producers and researchers), the samples were grouped as suggested by the producers. Samples were grouped as follows: 32 spoiled and 45 non-spoiled. This grouping was used for statistics to cluster microbiological data from brines, as shown in Figure 1.

Figure 1A reports the TAC count in the brines, with mean values of 7.25 ± 0.21 log CFU/mL (mean ± standard error) in the spoiled samples and 6.03 ± 0.27 log CFU/mL in the non-spoiled samples. The difference was significant (*t*-test, *p* < 0.05); LAB level was 5.79 ± 0.19 log CFU/mL in the non-spoiled samples and 4.30 ± 0.28 log CFU/mL in the spoiled samples, as shown in Figure 1B.

In order to see if it is possible to use TAC and LAB to describe spoiled and non-spoiled samples, the ratio LAB/TAC was analyzed as an index of the qualitative composition of bacterial microbiota (aerobic vs. lactic acid bacteria), as shown in Figure 1C. The ratio was evaluated by using the exponential values of cell counts to avoiding a possible “masking” exerted by logarithmic values. In spoiled samples this index was 0.26% (0.13–0.39%, 95%-confidence interval) thus suggesting that, in these batches, bacterial microbiota were mainly composed of aerobic microorganisms—in fact, their count was at least 99% higher than LAB. On the other hand, in the non-spoiled samples the ratio of LAB/TAC was 184%, with a confidence interval of ±181%.

Yeasts were 5.87 ± 0.19 log CFU/mL in the spoiled samples and 3.68 ± 0.28 log CFU/mL in the non-spoiled samples, as shown in Figure 1D. Enterobacteria were generally below the detection limit and they were found in only five samples with concentrations ranging from 1.21 to 2.09 log CFU/mL. Clostridia, bacilli, and pseudomonads were found only in 3–6 samples (ca. 1 log CFU/mL. Staphylococci were found in most samples and were at 2.09 ± 0.45 log CFU/mL and 2.42 ± 0.38 log CFU/mL in the spoiled and non-spoiled samples, respectively; the difference was not significant (*t*-test, *p* > 0.05), as shown in Figure 1E.

NaCl content varied from 6 to 8.5%; the pH was 4.2 ± 0.3 in the non-spoiled samples and 4.6 ± 0.6 in the spoiled samples (*t*-test, *p* > 0.05).

### 3.2. MoS

As a result of the microbiological analyses of the first phase, a user-friendly spreadsheet was prepared on Microsoft Excel—the spreadsheet was called MoS. The main idea was a spreadsheet where a user could input his/her own values on the quality and technological parameters in brines (TAC, LAB, yeasts, staphylococci, and pH) and preview the classification of the batch’s quality.

**Figure 2:**
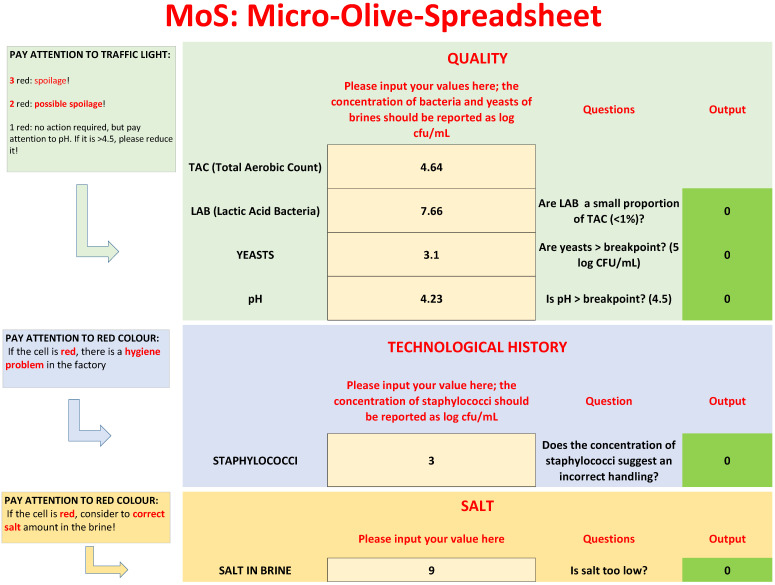
Input and Output of MoS.

The spreadsheet is organized in three parts: (a) microbiological quality; (b) technological history; (c) salt, as shown in Figure 2. Users can only add values in the yellow cells reading, “Please input your values here”. The other sections of spreadsheet are protected.

The main outputs of the spreadsheet are a preliminary evaluation of microbiological quality and a focus on the technological history of a batch to point out an incorrect handling of olives.

**Figure 3:**
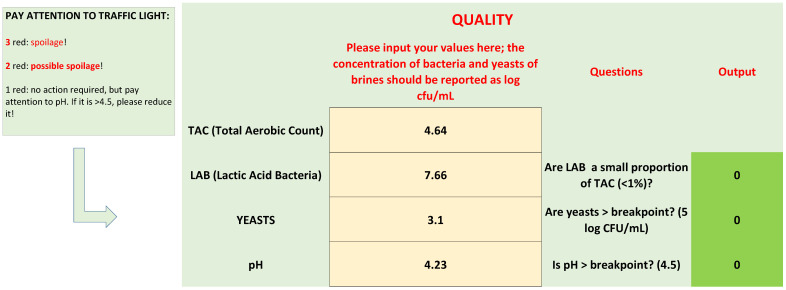
Section of spreadsheet for quality parameters.

Microbiological quality is based upon three criteria: the difference between TAC and LAB, yeast count, and pH. Each criterion is reported as a question, as shown in Figure 3:

1. Are LAB a small proportion of TAC in brines? This criterion was written as follows: LAB/TAC < 1%; both TAC and LAB in the equation were used as exponential values. However, user does not convert his/her values, because there is a function set in the protected cell of the “If” function, as shown in Table 1.
2. Are yeasts higher than the break-point? The threshold for yeasts in brines was set to 5 log CFU/mL.
3. The last criterion was on pH; although the results of the first phase did not show a clear difference between the spoiled and non-spoiled samples, a criterion on pH was added because of its role in the beginning of microbiological spoilage in the post-fermentation phase.

After entering the values of TAC, LAB, yeasts, and the pH of brine, MoS answers the three questions, with one of two codes: 0 for “no”, and 1 for “yes”. To help users understand the impact of the answers, a visual code was added, like a traffic-light. If the answer is yes (hazard), the cell assumes a the color red, while for 0 (no risk), the cell becomes green.

Legends show the key for the correct evaluation of the results:

1. Three red cells: There is probably microbiological spoilage.
2. Two red cells: A correction strategy is required, because a spoilage could start or have started.
3. One red cell: No spoilage and no action required; however, advice was added for pH. If pH is >breakpoint, reduce it to the break-point or, better, to 4.3.

**Figure 4:**
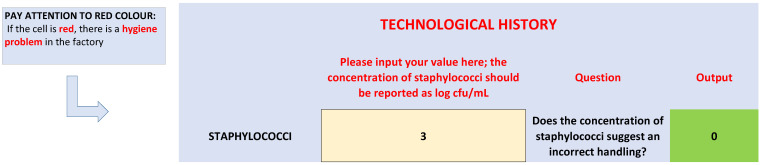
Section of spreadsheet on technological history.

The second section of the worksheet, as shown in Figure 4, is a focus on the technological history of olives; staphylococci are generally indicator microorganisms, mainly for use in GMP (Good Manufacturing Practices).

The results of the first phase showed that staphylococci could be a significant part of the bacterial microbiota of brines. For this section, an arbitrary threshold was set, because the advice is that a high number of these microorganisms could be the result of incorrect handling; the breakpoint was set to 4 log CFU/mL. Staphylococci do not play a role in the definition of the microbiological quality, according to the criteria reported above. They are hygiene indicators and are normally transferred to olives by food handlers. The break point (4 log CFU/mL) could be modified to fit HACCP plans and regional regulations; however, the meaning for this last criterion is the following: if staphylococci count is higher than the threshold (4 log CFU/mL, as in this paper, or lower if required by other regulations) corrective measures are required because there is a serious hygiene problem in the factory.

**Figure 5:**
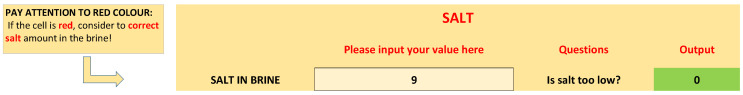
Section of spreadsheet for salt concentration in brine.

The last section of MoS is on salt, as shown in Figure 5. The analysis of the results of the first part did not show a significant difference between spoiled and non-spoiled samples. However, a criterion on salt was added as advice for producers to perform corrective measures when NaCl <8%.

### 3.3. Validation

**Figure 6:**
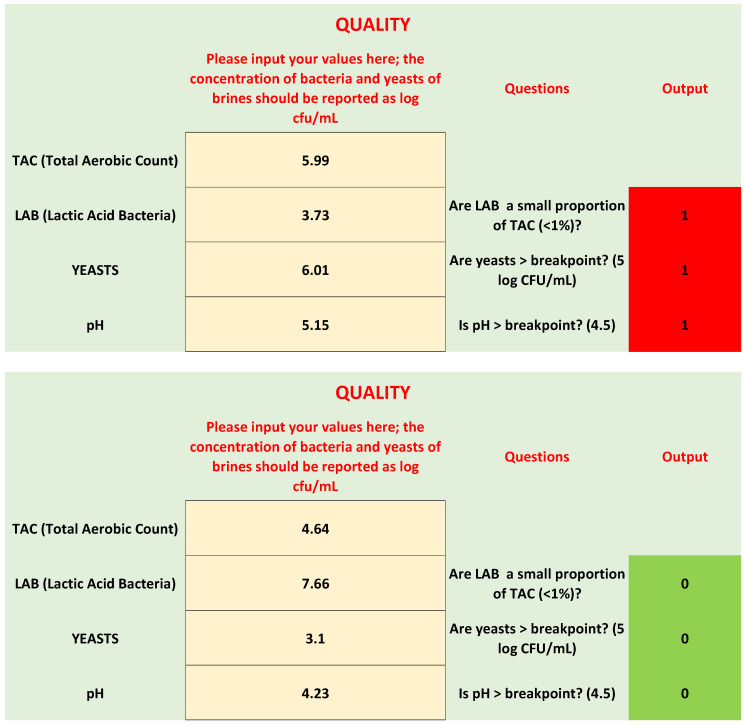
Traffic light for spoiled (sample 2) and acceptable samples (sample 6).

Twelve samples, not used for the spreadsheet design (Section 3.1 and Section 3.2), were used for a preliminary validation. They were randomly selected after their collection in the factories (three per factory) and analyzed as reported above for the sensory scores and microbiology. The results are in Table 2. Figure 6 shows the output of the spreadsheet for two samples.

Five samples (1, 6, 8, 9, and 12) were recorded as acceptable or non-spoiled by the panelists (both producers and researchers). The combination of parameters on the spreadsheet (ratio of LAB/TAC, yeast count, and pH) gave the same result. Samples 2, 3, 10, and 11 were judged as not-acceptable or spoiled by the panelists. Generally, the spreadsheet returned the same output, except for sample 3, because two parameters (yeast count and pH) were in the hazard zone, while the ratio of LAB/TAC was lower than 1%. Finally, samples 4, 5, and 7 were recorded as doubtful samples by the panelists and were included in the attention class by the spreadsheet, because at least two parameters were out of range.

For all of the samples NaCl was recorded as acceptable by the producers (8–10%), enterobacteria and pseudomonads were always below the detection limit, and bacilli and clostridia were found only in two samples, but their level was very low (1–1.2 log CFU/mL). 

Staphylococci were found in almost all samples, but their level was <2 log CFU/mL in brines, except for the samples 3, 5, 10, and 11, showing a count of staphylococci in the range 3.2–4.1 log CFU/mL; coagulase positive staphylococci were found in samples 3 and 4, both within range, according to the spreadsheet.

## 4. Discussion

The post-fermentation stage is a critical step for the production of table olives, as they are usually stored in tanks filled with brine for several months and, when the temperature increases (February and March), spoilage can occur. 

Predictive microbiology is experiencing an increase in interest by olive producers, because they need tools to predict olive shelf life and/or to act with corrective measures when problems occur. An interesting example of a predictive tool is the Decision-Making System for Safety and Quality Management in Aloreña de Málaga [25]. Other applications of predictive microbiology rely upon the use of the theory of Design of Experiments or neural networks (among others [13,26]). However, during the project BiotecA we met several producers of Table Olives of Apulia and they shared with us the idea of a user-friendly spreadsheet where they could enter the values of brine to understand the microbiological scenario.

The idea is to develop this project into a tool similar to Risk Ranger [27], which is an educational and research tool developed by Australia Food Safety. Users have to answer some questions related to the technology, preparation, cooking, and storage of a food—the output is a risk rank (from 0 to 100). The benefit of this kind of tool is that it works in a spreadsheet with an Excel interface (user-friendly) and the output is understandable for non-expert users.

The first part of this research was aimed at understanding if there was a connection between the idea of spoilage by producers and the microbiological profile of olives. The preliminary results showed a link with some indices, such as yeasts, pH, and the ratio of LAB/TAC. Yeasts exert a dual role in table olives—they can cause spoilage due to the production of CO_2_, bad odors and flavors, the clouding of brines or the softening of fruits [5,28,29]. In the post-fermentation stage, oxidative yeasts, such as *Pichia anomala* and *P. membranifaciens*, could prevail. In addition, film-forming yeasts (*Debaryomyces*, *Candida*, *Pichia,* and *Endomycopsis*) are often associated with pickled products and vegetable brines [30], representing the cause of olive defects and consequent product losses. Salt is the main factor able to control the yeasts and LAB during olive fermentation [31]. Yeasts dominate fermentations at salt levels > 10% NaCl; however, this process leads to a final product with a milder taste and less self-preservation characteristics [31], while salt reduction to 6–8% enables a mixed fermentation by lactic acid bacteria and yeasts that coexist until the end of fermentation, resulting in a product with better characteristics [32]. However, this practice, often used by Bella di Cerignola producers, could be responsible for the survival and/or growth of some oxidative yeasts, as reported by Fuccio et al. [5]. The analysis performed in the screening step suggested that yeasts could play a negative role in olive quality; therefore, they were set as a negative criterion in MoS. To the best of our knowledge, there is no evidence on the critical threshold of yeasts on vegetables; however, the breakpoint in MoS was set to 5 log CFU/mL because this was related to spoiled samples and is the critical threshold associated with spoilage in many foods [33].

The analyses of the first part also suggested a link between spoilage and the ratio of LAB vs. TAC. Aerobic plate counts are poor indicators of safety in some products, such as those that are fermented, which commonly show a high aerobic count. However, TAC gives information about the hygienic and sensorial quality, the adherence to good manufacturing practice, and the shelf life of the product [7,34]. The link of the ratio of LAB/TAC with the spoilage suggests that the negative effects of some aerobic bacteria could be counteracted by LAB [3,7]. TAC in table olives include *Bacillus* spp., *Aerobacter* spp., and *Pseudomonas* spp. All of these bacteria, along with some fungi, release degrading enzymes, which act on pectic substances and cellulose, hemicellulose, and polysaccharides, causing the loss of the structural integrity of the olive drupe [21,35]. 

pH is a key variable for olive safety and quality. An incorrect acidification of brine is a common problem for small farms where fermentation takes place without the use of starter cultures and the final pH of brine is around 5.0 [8]. Codex Alimentarius standard [36] sets the breakpoint for olive safety at 4.3; however, a pH of 4.5 is generally accepted by producers as safe, at least during storage in tanks. Another factor to control during the post-fermentation stage is salt. Although, the differences between spoiled and non-spoiled samples were not significant, salt could play a crucial role in the post-fermentation stage, mainly in spring when the increase in temperature requires additional corrective or control measures. Salt level in brines was at 6–8%; this amount could assure correct lactic fermentation and the dominance of LAB in the first stages. However, it could not be enough in post-fermentation to protect olives from abnormal fermentations [37], therefore, advice (third section) to perform corrective measure was added to MoS.

Eventually, the worksheet could be improved by adding in a revised version salt as a primary criterion; however, this criterion, as well as the advice of lowering pH to 4.3 or, better, to 4.1 to assure the microbiological stability of olives, could be the result of some changes in the habits of olive producers in Apulia. 

Therefore, as a preliminary step the spreadsheet was developed for yeasts, LAB/TAC, and pH at 4.5 and the preliminary validation showed a good agreement between spoilage status, as revealed by the producers, and microbiological profile. Further investigations are required for a validation on batches from other olive producers, as well as for other olive varieties. Moreover, it was not possible to link the quality with other genera/groups of microorganisms, because in our samples they were detected occasionally.

Finally, another criterion we suggest for quality assessment is staphylococci count. In many foods the presence of staphylococci usually indicates post-processing contamination from human skin, mouths, and noses, or food handlers [38]. Due to their high salt tolerance, they can grow in table olives despite the low pH and the olive phenols may represent natural inhibitors [32].

In conclusion, this paper represents a first structured approach to design a user-friendly spreadsheet for the quick evaluation of the microbiological profile and quality of Bella di Cerignola olives during their storage in tanks. The spreadsheet is based upon four criteria (TAC, LAB, yeasts, and pH) and has two main benefits: (i) it is user-friendly; (ii) it gives an output on possible spoiling events in brines. In addition, the use of staphylocci as an indicator of microorganisms offers the possibility of analyzing and highlighting possible incorrect handling. Some issues to be addressed for an effective scaling up of the worksheet are the following: (a) The tool has been designed as an Excel spreadsheet, because this is the common software suite used by producers; however, the tool should be also designed in Apple Numbers, Open Office, or Google Sheet for a wide application; (b) It is based on lab data. The latter are not mandatory, but producers of the Apulia Region usually obtain them from experts or laboratories once or twice a month. The tool could be modified by adding screening criteria physico–chemical parameters (pH, salt, and temperature), available for producers many times per week, and microbiological counts as indices for the confirmatory classification of samples; (c) Safety is not a criterion in the tool, because producers of the Apulia region rarely have these data, since they are time-consuming and expensive, but the worksheet could be improved by pointing out some indicators (physico–chemical or microbiological) linked to safety. 

The spreadsheet could be modified by adding other criteria or by setting different breakpoints, depending on regional and/or national regulations, as well as on the HACCP plan of each producer; however, it is a simple tool for an effective application of Hazard Analysis and Predictive Microbiology in table olive production and to improve a sector with many critical points. 

The tool was developed for Bella di Cerignola olives, but a similar approach could be used to design a general tool for olives and to improve their performances by entering results from different seasons and places. 

## Figures and Tables

**Table 1:** Decision criteria for MoS (Micro-Olive-Spreadsheet) and respective equations and outcomes.

Decision Criteria (Questions)	Equations	Yes	No
**Quality**			
*Are LAB a small proportion of bacterial microbiota?*	LAB < 1% TAC	Not acceptable	Acceptable
*Is yeast concentration > breakpoint?*	Yeast > 5	Not acceptable	Acceptable
*Is pH > breakpoint?*	pH > 4.5	Not acceptable	Acceptable
**Technological Process**			
*Is handling incorrect?*	Staph > 4	Incorrect handling	-
*Is salt too low?*	Salt < 8%	Possible corrective measures	-

TAC, total aerobic count; LAB, lactic acid bacteria; staph, staphylococci. Bacterial concentrations of brines are reported as log CFU/mL.

**Table 2:** Decision on the samples used for the validation.

Sample	Decision by Panel	Comments	TAC	LAB	Yeasts	pH	Spreadsheet
1	Acceptable	Typical odor of Spanish-style olives	2.01	6.65	4.18	4.05	Acceptable
2	Not acceptable	Off-odors	5.99	3.73	6.01	5.15	Not acceptable
3	Not acceptable	Off-odors	6.14	5.93	6.14	5.27	Attention
4	Acceptable, but the sample has some problems	Films on the surface	5.45	5.08	7.26	5.35	Attention
5	Acceptable, but the sample has some problems	Strong odor	5.53	2.23	3.46	4.67	Attention
6	Acceptable	-	4.64	7.66	3.10	4.23	Acceptable
7	Acceptable, but the sample has some problems	Fruity odor is too strong	4.82	7.63	5.87	4.85	Attention
8	Acceptable	-	4.56	6.12	4.53	4.43	Acceptable
9	Acceptable	-	4.31	5.99	3.56	4.36	Acceptable
10	Not acceptable	Films on the surface	7.80	5.79	6.01	4.67	Not acceptable
11	Not acceptable	Off-odors	5.99	2.34	5.31	4.89	Not acceptable
12	Acceptable	-	5.53	6.48	2.83	4.43	Acceptable

TAC, total aerobic count; LAB, lactic acid bacteria. The unit of counts is log CFU/mL.

## Supplementary Materials

The following are available online at https://www.mdpi.com/2304-8158/9/7/848/s1, Spreadsheet S1: MoS (Micro-Olive-Spreadsheet).

## References

[1] Benítez-Cabello A., Romero-Gil V., Medinaa E., Sánchez B., Calero-Delgado B., Bautista-Gallego J., Jiménez-Díaz R., Arroyo-López F.N. (2019). Metataxonomic analysis of the bacterial diversity in table olive dressing components. Food Control.

[2] Mastralexi A., Mantzouridou F.T., Tsimidou M.Z. (2019). Evolution of safety and other quality parameters of the Greek PDO table olives “Prasines Elies Chalkidikis” during industrial scale processing and storage. Eur. J. Lipid Sci. Technol..

[3] Perpetuini G., Prete R., Garcia-Gonzalez N., Alam M.K., Corsetti A. (2020). Table olives more than a fermented food. Foods.

[4] Randazzo C.L., Todaro A., Pino A., Pitino I., Corona O., Caggia C. (2017). Microbiota and metabolome during controlled and spontaneous fermentation of Nocellara Etnea table olives. Food Microbiol..

[5] Fuccio F., Bevilacqua A., Sinigaglia M., Corbo M.R. (2016). Using a polynomial model for fungi from table olives. Int. J. Food Sci. Technol..

[6] Nychas G.J.E., Panagou E.Z., Parker M.L., Waldron K.W., Tassou C.C. (2002). Microbial colonization of naturally black olives during fermentation and associated biochemical activities in the cover brine. Lett. Appl. Microbiol..

[7] Romeo F.V., Muzzalupo I. (2012). Microbiological aspects of table olives. Olive Germplasm. The Olive Cultivation, Table Olive and Olive Oil Industry in Italy.

[8] Perricone M., Bevilacqua A., Corbo M.R., Sinigaglia M. (2010). Use of *Lactobacillus plantarum* and glucose to control the fermentation of “Bella di Cerignola” table olives, a traditional variety of Apulian region (Southern Italy). J. Food Sci..

[9] Bevilacqua A., Cannarsi M., Gallo M., Sinigaglia M., Corbo M.R. (2010). Characterization and implications of *Enterobacter cloacae* strains, isolated from Italian table olives “Bella di Cerignola”. J. Food Sci..

[10] Panagou E.Z., Nychas G.J.E., Sofos J.N. (2013). Types of traditional Greek foods and their safety. Food Control.

[11] Medina-Pradas E., Arroyo-López F.N. (2015). Presence of toxic microbial metabolites in table olives. Front. Microbiol..

[12] Tataridou M., Kotzekidou P. (2015). Fermentation of table olives by oleuropeinolytic starter culture in reduced salt brines and inactivation of *Escherichia coli* O157:H7 and *Listeria monocytogenes*. Int. J. Food Microbiol..

[13] Bevilacqua A., Campaniello D., Speranza B., Sinigaglia M., Corbo M.R. (2018). Survival of *Listeria monoctytogenes* and *Staphylococcus aureus* in synthetic brines. Studying the effects of salt, temperature and sugar through the approach of the Design of the Experiments. Front. Microbiol..

[14] Abriouel H., Benomar N., Lucas R., Gálvez A. (2011). Culture-independent study of the diversity of microbial populations in brines during fermentation of naturally fermented Aloreña green table olives. Int. J. Food Microbiol..

[15] Lavermicocca P., Angiolillo L., Lonigro S.L., Valerio F., Bevilacqua A., Perricone M., Del Nobile M.A., Corbo M.R., Conte A. (2018). *Lactobacillus plantarum* 5BG survives during the refrigerated storage bio-preserving packaged Spanish-style table olives (cv. Bella di Cerignola). Front. Microbiol..

[16] Comunian R., Ferrocino I., Paba A., Daga E., Campus M., Di Salvo R., Cauli E., Piras F., Zurru R., Cocolin L. (2017). Evolution of microbiota during spontaneous and inoculated Tonda diCagliari table olives fermentation and impact on sensory characteristics. LWT-Food Sci. Technol..

[17] Randazzo C.L., Todaro A., Pino A., Pitino I., Corona O., Mazzaglia A., Caggia C. (2014). Giarraffa and Grossa di Spagna naturally fermented table olives: Effect of starter and probiotic cultures on chemical, microbiological and sensory traits. Food Res. Int..

[18] D’Antuono I., Bruno A., Linsalata V., Minervini F., Garbetta A., Tufariello M., Mita G., Logrieco A.F., Bleve G., Cardinali A. (2018). Fermented Apulian table olives: Effect of selected microbial starters on polyphenols composition, antioxidant activities and bioaccessibility. Food Chem..

[19] Bleve G., Tufariello M., Durante M., Perbellini E., Ramires F.A., Grieco F., Cappello M.S., De Domenico S., Mita G., Tasioula-Margari M. (2014). Physico-chemical and microbiological characterization of spontaneous fermentation of Cellina di Nardò and Leccino table olives. Front. Microbiol..

[20] Lucena-Padrós H., Ruiz-Barba J.L. (2019). Microbial biogeography of Spanish-style green olive fermentations in the province of Seville, Spain. Food Microbiol..

[21] Lanza B. (2013). Abnormal fermentations in table olive processing: microbial origin and sensory evaluation. Front. Microbiol..

[22] Bevilacqua A., de Stefano F., Augello S., Pignatiello S., Sinigaglia M., Corbo M.R. (2015). Biotechnological innovations for table olives. Int. J. Food Sci. Nutr..

[23] Campaniello D., Bevilacqua A., D’Amato D., Corbo M.R., Altieri C., Sinigaglia M. (2005). Microbial characterization of table olives processed according to Spanish and Natural styles. Food Technol. Biotechnol..

[24] International Olive Oil Council (IOC) Method. Sensory Analyses of Table Olives. COI/OT/MO No 1/Rev.2, November 2011. https://www.internationaloliveoil.org/what-we-do/chemistry-standardisation-unit/standards-and-methods/.

[25] Ruiz-Bellido M.Á., Valero A., Medina Pradas A., Romero Gil V., Rodríguez-Gómez F., Posada-Izquierdo G.D., Rincón F., Possas A., García-Gimeno R.M., Arroyo-López F.N. (2017). A probabilistic decision-making scoring system for quality and safety management in Aloreña de Málaga table olive processing. Front. Microbiol..

[26] Panagou E.Z., Kodogiannis V., Nychas G.J.-E. (2007). Modelling fungal growth using radial basis function neural networks: The case of the ascomycetous fungus *Monascus ruber* van Tieghem. Int. J. Food Microbiol..

[27] Risk Ranger. https://www.cbpremium.org/RiskRanger.

[28] Bevilacqua A., Beneduce L., Sinigaglia M., Corbo M.R. (2013). Selection of yeasts as starter cultures for table olives. J. Food Sci..

[29] Bevilacqua A., Corbo M.R., Sinigaglia M. (2012). Selection of yeasts as starter cultures for table olives: A step-by-step procedure. Front. Microbiol..

[30] Fleming H.P., Mcfeeters R.F., Breidt F., Downes F.P., Ito K. (2001). Staphylococcus aureus and staphyolococcal enterotoxins. Compendium of methods for the Microbiological examination of Foods.

[31] Leventdurur S., Sert-Aydın S., Boyaci-Gunduz C.P., Agirman B., Ben Ghorbal A., Francesca N., Martorana A., Erten H. (2016). Yeast biota of naturally fermented black olives in different brines made from cv. Gemlik grown in various districts of the Cukurova region of Turkey. Yeasts.

[32] Tassou C.C., Nychas G.J.E. (1994). Inhibition of *Staphylococcus aureus* by olive phenolics in broth and in a model food system. J. Food Prot..

[33] Perricone M., Gallo M., Corbo M.R., Sinigaglia M., Bevilacqua A., Bevilacqua A., Corbo M.R., Sinigaglia M. (2017). Yeasts. The microbiological quality of food. Foodborne spoilers.

[34] Morton R.D., Downes F.P., Ito K. (2001). Aerobic plate count. Compendium of Methods for the Microbiological Examination of Foods.

[35] Golomb B.L., Morales V., Jung A., Yau B., Boundy-Mills K.L., Marco M.L. (2013). Effects of pectinolytic yeast on the microbial composition and spoilage of olive fermentations. Food Microbiol..

[36] (2013). CODEX/COI Codex Standard for Table Olives. CODEX STAN 66-1881. Revision 1987.

[37] Garrido-Fernández A., Fernández-Díez M.J., Adams R.M. (1997). Table Olives: Production and Processing.

[38] Lancette G.A., Bennett R.W., Downes F.P., Ito K. (2001). Fermented and acidified vegetables. Compendium of Methods for the Microbiological Examination of Foods.

